# Equivalence between physicians and associate clinicians in the frequency of iatrogenic urogenital fistula following cesarean section in Tanzania and Malawi

**DOI:** 10.1186/s12960-024-00927-8

**Published:** 2024-06-24

**Authors:** Carrie J. Ngongo, Thomas J. I. P. Raassen, Jos van Roosmalen, Marietta Mahendeka, Ladeisha Lombard, Elizabeth Bukusi

**Affiliations:** 1 RTI International, Global Health Division, Research Triangle Park, United States of America; 2 Independent Consultant, Weesp, the Netherlands; 3 Leiden University Medical Centre and Athena Institute VU University, Amsterdam, Netherlands; 4 Bugando Medical Centre, Mwanza, Tanzania; 5 Independent Consultant, Cape Town, South Africa; 6 Research Care Training Program in the Center for Microbiology Research, KEMRI, Nairobi, Kenya; 7 Departments of Global Health and Obstetrics and Gynecology, University of Washington, Seattle, United States of America; 8 Department of Obstetrics and Gynecology, University of Nairobi, Nairobi, Kenya

**Keywords:** Human resources for health, Task shifting, Associate clinicians, Cesarean section, Iatrogenic fistula

## Abstract

**Background:**

Physicians and associate (non-physician) clinicians conduct cesarean sections in Tanzania and Malawi. Urogenital fistulas may occur as complications of cesarean section. Location and circumstances can indicate iatrogenic origin as opposed to ischemic injury following prolonged, obstructed labor.

**Methods:**

This retrospective review assessed the frequency of iatrogenic urogenital fistulas following cesarean sections conducted by either associate clinicians or physicians in Tanzania and Malawi. It focuses on 325 women with iatrogenic fistulas among 1290 women who had fistulas after cesarean birth in Tanzania and Malawi between 1994 and 2017. An equivalence test compared the proportion of iatrogenic fistulas after cesarean sections performed by associate clinicians and physicians (equivalence margin = 0.135). Logistic regression was used to model the occurrence of iatrogenic fistula after cesarean section, controlling for cadre, date, maternal age, previous abdominal surgery and parity.

**Results:**

Associate clinicians attended 1119/1290 (86.7%) cesarean births leading to fistulas, while physicians attended 171/1290 (13.3%). Iatrogenic fistulas occurred in 275/1119 (24.6%) cesarean births by associate clinicians and in 50/171 (29.2%) cesarean births by physicians. The risk difference and 90% confidence interval were entirely contained within an equivalence margin of 13.5%, supporting a conclusion of equivalence between the two cadres. The odds of iatrogenic fistula after cesarean section were not statistically significantly different between associate clinicians and physicians (aOR 0.90; 95% CI 0.61–1.33).

**Conclusions:**

Associate clinicians appear equivalent to physicians performing cesarean sections in terms of iatrogenic fistula risk. Lower iatrogenic proportions for associate clinicians could reflect different caseloads. The occurrence of iatrogenic fistulas illustrates the importance of appropriate labor management and cesarean section decision-making, irrespective of health provider cadre. Given the noninferior performance and lower costs of employing associate clinicians, other countries with insufficient and/or unequally distributed health workforces could consider task-shifting cesarean sections to associate clinicians.

## Background

Two-thirds of global maternal deaths occur in sub-Saharan Africa, largely due to preventable and treatable complications such as hypertensive disorders, obstetric hemorrhage and maternal sepsis [1, 2]. Ensuring maternal and neonatal survival requires comprehensive emergency obstetric and newborn care for mothers experiencing life-threatening complications during childbirth [3]. Comprehensive emergency obstetric and newborn care encompasses basic obstetric care as well as safe blood transfusion, advanced newborn resuscitation and cesarean section.

When indicated, cesarean sections save maternal and newborn lives, particularly in countries with low cesarean section rates and high maternal mortality ratios [4, 5]. Compared with vaginal birth, cesarean sections are associated with greater risks of maternal and perinatal death and newborn sepsis as well as maternal morbidity from abnormal invasive placentation and uterine scar rupture in subsequent pregnancies [6–9]. In remote and under-resourced areas, the risks associated with cesarean birth differ significantly, with maternal mortality after cesarean birth being 50 times greater in sub-Saharan Africa compared to high-income settings [10]. Cesarean births constitute nearly one-third of all surgeries in resource-poor settings [11].

The availability of quality cesarean sections depends upon trained caregivers. Yet, the number of available healthcare workers is lowest in places where healthcare workers are most needed. Globally, the shortage of human resources for health is most acute in sub-Saharan Africa, where the World Health Organization reports that 36 countries face a critical shortage of doctors, nurses and midwives [12, 13]. Many countries are training increasing numbers of clinicians in response to the clear needs of growing populations. At national and sub-national levels, health personnel are concentrated in urban areas and are insufficiently distributed in rural, low-resource areas where maternal mortality and morbidity are highest [14].

To address the challenges in the number and distribution of physicians, many countries deploy associate clinicians, non-physicians who take on many of the diagnostic and clinical functions of doctors [15]. With various titles including assistant medical officers, clinical officers, health officers, medical assistants and physician assistants, associate clinicians were active in 25 of 47 sub-Saharan African countries in 2007 [15]. Tanzania and Malawi use task shifting to expand obstetric surgical capacity: associate clinicians are authorized and trained to perform cesarean sections and other major emergency obstetric surgeries [16]. They are two of six countries in sub-Saharan Africa where associate clinicians perform cesarean sections; others are Ethiopia, Ghana, Mozambique and Sierra Leone [15, 17, 18].

Associate clinicians have training beyond secondary school, typically with more clinical skills than nurses but fewer than doctors [15]. Associate clinician training focuses on diagnosis and medical treatment, including prescribing medications. Associate clinicians in Malawi and Tanzania typically do not have a university qualification before completing at least three years of medical training and being licensed to provide general medical care [15]. In contrast, physicians (medical officers) in Malawi and Tanzania complete 5 years of medical training and an internship in medicine, pediatrics, surgery and obstetrics/gynecology [19]. Physician training typically includes greater emphasis on basic anatomy, physiology and pathology, advanced technology and hospital-based care [15].

Task shifting represents a strategic response to substantial workforce volume and distribution challenges in both countries. In 2018, Tanzania had an estimated one physician for 17 544 people, while Malawi had one physician for 71 429 people [12]. Maternal mortality has declined substantially in both countries yet remains a persistent challenge. According to 2020 estimates from the World Health Organization, the maternal mortality ratio was 238 deaths per 100 000 live births in Tanzania and 381 deaths per 100 000 live births in Malawi [20].

Associate clinicians have been central to improve staffing for essential surgery in places where they are authorized and trained to provide comprehensive emergency obstetric and newborn care [21]. Although task shifting to associate clinicians can improve mothers’ access to emergency obstetric care, physicians in many countries remain skeptical about associate clinicians providing cesarean sections [15, 22]. Despite documentation regarding the feasibility and outcomes of task shifting for cesarean sections, this strategy has not been widely embraced by other countries experiencing human resource crises and high maternal mortality [21]. Ongoing evidence-generation and communication are therefore useful for informing policymakers who may be considering expanding the cadres that are authorized and trained to provide cesarean sections.

This review uses a data set of women who presented for urogenital fistula repair to consider the circumstances of fistula development. Urogenital fistulas may occur between any part of the genital tract and nearby organs in the pelvis, including the bladder or rectum. Women with urogenital fistulas suffer from urinary and/or fecal incontinence. Most urogenital fistulas are ischemic and are caused by pressure necrosis when women in neglected prolonged (obstructed) labor are not able to undergo emergency cesarean section in time. Fistulas can also be iatrogenic, arising as surgical accidents during obstetric or gynecological surgery. Cesarean birth is becoming more frequent as the mode of birth that is reported by women who have developed fistulas and sought fistula repair [23]. The occurrence of iatrogenic fistulas during cesarean birth is a sentinel event reflecting the overall quality of surgical care [24, 25]. A variety of outcomes could be used to assess cesarean section quality. Given the availability of retrospective data from women seeking fistula repair, this analysis compares the frequency of iatrogenic urogenital fistulas following cesarean section performed by physicians and associate clinicians in Tanzania and Malawi.

## Methods

This retrospective records review evaluated the frequency of cesarean-associated iatrogenic fistulas by cadre among 1290 women who presented with fistula-related incontinence after cesarean birth in Tanzania and Malawi. These women are a subset of a larger group of 6787 women who presented for fistula repair in nine countries [25, 26]. As detailed elsewhere, TJIPR, MM, and their colleagues collected data between June 1994 and December 2017 [27]. One of the surgeons interviewed the women and recorded information on a standard form, documenting the woman’s age at presentation, age at fistula development, obstetric history (including previous births and previous laparotomies) and mode of birth [27]. Women provided information about the condition of the newborn at birth. The operating surgeon noted the fistula’s classification based on Waaldijk’s classification system, which categorizes fistulas based on their anatomic/physiologic position, involvement of the closing mechanism and urethra, and whether the damage is circumferential [28]. TJIPR examined all women and reviewed and verified all fistula classifications and operations.

Women seeking fistula repair in Tanzania and Malawi were interviewed in 36 facilities, largely district and mission hospitals. Two-thirds of these facilities where fistulas were repaired were in rural areas. Women had developed fistulas following cesarean section in an unknown, larger number of hospitals some time before seeking repair. Detailed information regarding these facilities is not available.

TJIPR noted the cadre of the health provider who performed the cesarean section on the basis of the description provided by the woman and his knowledge of local facilities and staffing, which was complemented by input from local staff. This analysis groups all physicians, including medical officers and those with additional training, such as registrars (residents) and specialists (who have completed residency in obstetrics and gynecology). Associate clinicians are called assistant medical officers in Tanzania and clinical officers in Malawi.

Women seeking fistula repair in Tanzania or Malawi from TJIPR, MM and colleagues were eligible for inclusion in this analysis if they had a fistula following cesarean section, cesarean hysterectomy or uterine rupture repair, grouped together as “cesarean birth.” All cesareans were emergency procedures that occurred during labor. Women were excluded if they developed fistulas following vaginal birth, gynecological surgery, traumatic injury (from accidents, traditional healers or sexual violence), abortion or radiation. Women were also excluded if their causative births occurred before 1994.

Fistula origin can be ambiguous in cases where women labor for a long time before cesarean section. This analysis follows previously proposed definitions for the likelihood of iatrogenic etiology [19]. “Definitely iatrogenic” fistulas occur away from where fetal head pressure against the mother’s pelvis would lead to ischemia and necrosis. This category includes ureteric injuries, which arise from an inadvertent nick, cut or tie of the distal ureter near the cervix where it is crossed by uterine vessels [29]. This category also includes vesico-cervico-vaginal fistulas following the cesarean birth of a live baby, as the cesarean birth of a live baby is rarely associated with pressure necrosis. “Probably iatrogenic” fistulas further include vault fistulas following cesarean-hysterectomy. A vault fistula is a connection between the bladder and the top of the vagina after total removal of the uterus, most often for a ruptured uterus. However, fistulas should not be considered iatrogenic in cases where bladder rupture occurs. “Likely iatrogenic” fistulas further include vesico-cervico-vaginal fistulas following cesarean birth with stillbirth, as long as the fistulas are small (< 3 cm), clearly in the cervical canal, and without bladder rupture. Fistulas not meeting the above criteria were not considered to be iatrogenic. TJIPR applied these criteria to determine the origin of fistulas for all included records on the basis of his examination and the surgical notes. A previous analysis applied these definitions to assess the rise in iatrogenic fistulas after cesarean section in nine African countries [25].

Data were entered into an Excel database, with names changed to unique identification numbers to protect privacy. Data were analyzed using Stata 16 software (StataCorp). Country-specific logistic regressions were used to model iatrogenic fistulas among cesarean births resulting in fistula by cadre (physicians and associate clinicians). The models included the year of fistula development, age at fistula development, parity and previous abdominal surgery (nearly always a cesarean birth). We used categories for fistula development dates (1994–1999 as a reference, 2000–2004, 2005–2009 and 2010–2017), parities (1, 2, 3–5, 6+), and age at fistula development (11–19 as a reference, 20–24, 25–29, 30–34, 35+). The threshold for statistical significance was *p* < 0.05.

Equivalence testing is more appropriate than traditional statistical tests of differences (such as ANOVA and *t* tests) in cases where the goal is to demonstrate similarity between approaches. Equivalence testing reverses the traditional null and alternative hypotheses, such that the null hypothesis is that the two approaches are not equivalent [30, 31]. In this case, our null hypothesis was that the difference in the risk of iatrogenic fistula after cesarean birth was not equivalent between associate clinicians and physicians, as measured by a generalized linear model. The equivalence region, δ, was defined as a 13.5% difference in means in iatrogenic fistula occurrence between cesarean births performed by associate clinicians and physicians. An observed difference within this definition would therefore be considered evidence of equivalence between the two cadres. Equivalence testing involves two one-sided tests (TOSTs), each carried out at a statistical significance level of *p* < 0.05. As such, the confidence interval was (1-2α) × 100% or 90%.

## Results

Associate clinicians performed 1119/1290 (86.7%) of the cesarean births leading to fistulas, including 86.0% (833/969) in Tanzania and 89.1% (286/321) in Malawi (Table 1). One-quarter of genital fistulas following cesarean birth were iatrogenic in both Tanzania (242/970, 25.0%) and Malawi (83/321, 25.9%). An iatrogenic fistula occurred in 275/1119 (24.6%) of cesarean births by associate clinicians compared to 50/171 (29.2%) of cesarean births by physicians (Table 2). Stillbirths occurred in 1035/1287 (80.4%) of the cesarean births leading to fistulas.

**Table 1:** Cadre attending cesarean birth leading to fistulas

	Cadre attending birth		Total
	Associate clinician	Physician	
Tanzania	833	136	969
	86.0%	14.0%	100%
Malawi	286	35	321
	89.1%	10.9%	100%
Total	1119	171	1290
	86.7%	13.3%	100%

**Table 2:** Iatrogenic fistula occurrence by attending cadre

	Cadre attending birth		Total
	Associate clinician	Physician	
Not iatrogenic	844	121	965
	75.4%	70.8%	74.8%
Iatrogenic	275	50	325
	24.6%	29.2%	25.2%
Total	1119	171	1290
	100%	100%	100%

Combined and country-specific logistic regressions are provided in Table 3. Controlling for date, age, parity and previous abdominal surgery, the odds of iatrogenic fistula after cesarean section were not statistically significantly different between associate clinicians and physicians, neither overall (aOR 0.90; 95% CI 0.61–1.33) nor in Tanzania (aOR 0.80; 95% CI 0.51–1.24) nor in Malawi (aOR 1.44; 95% CI 0.65–3.17).

**Table 3:** Logistic regression of iatrogenic fistulas among cesarean births resulting in fistulas

		Overall			Tanzania			Malawi		
		*n* = 1113			*n* = 964			*n* = 318		
	*n*	Odds ratio	95% confidence interval		Odds ratio	95% confidence interval		Odds ratio	95% confidence interval	
Cadre										
Associate clinician	1119	(Ref)	(Ref)		(Ref)	(Ref)		(Ref)	(Ref)	
Physician	171	0.90	0.61	1.33	0.80	0.51	1.24	1.44	0.65	3.17
Date of fistula development										
1994–1999	211	(Ref)	(Ref)		(Ref)	(Ref)		(Ref)	(Ref)	
2000–2004	421	**0.22**	**0.16**	**0.29**	**0.22**	**0.16**	**0.30**	**0.23**	**0.11**	**0.48**
2005–2009	333	**0.19**	**0.13**	**0.26**	**0.20**	**0.14**	**0.29**	**0.14**	**0.07**	**0.29**
2010–2017	326	**0.22**	**0.16**	**0.31**	**0.18**	**0.12**	**0.28**	**0.27**	**0.16**	**0.46**
Previous abdominal surgery										
No	1145	(Ref)	(Ref)		(Ref)	(Ref)		(Ref)	(Ref)	
Yes	144	**2.48**	**1.67**	**3.69**	**2.83**	**1.80**	**4.48**	1.78	0.77	4.08
Parity										
1	492	(Ref)	(Ref)		(Ref)	(Ref)		(Ref)	(Ref)	
2	195	**2.36**	**1.49**	**3.72**	**2.50**	**1.45**	**4.29**	2.10	0.85	5.15
3–5	348	**2.58**	**1.57**	**4.24**	**2.44**	**1.33**	**4.46**	**2.87**	**1.17**	**7.07**
6+	254	**4.20**	**2.29**	**7.70**	**4.47**	**2.14**	**9.33**	**3.93**	**1.29**	**11.92**
Age at fistula development										
11–19	383	(Ref)	(Ref)		(Ref)	(Ref)		(Ref)	(Ref)	
20–24	312	**0.41**	**0.27**	**0.62**	**0.38**	**0.23**	**0.63**	0.48	0.21	1.12
25–29	256	0.61	0.37	1.01	0.58	0.32	1.07	0.69	0.27	1.77
30–34	153	0.74	0.40	1.34	0.72	0.34	1.49	0.67	0.23	1.99
35+	183	0.69	0.36	1.30	0.70	0.32	1.51	0.64	0.20	2.05

Bold font indicates statistical significance

The factors associated with iatrogenic fistula etiology after cesarean section were directionally similar between Tanzania and Malawi, although they were more often statistically significant in Tanzania than Malawi due at least in part to Tanzania’s larger sample size. While the odds of iatrogenic origin among cesarean births resulting in fistula remained steady between 1994 and 2017 according to the 2 × 2 comparison, the date of fistula development was statistically significant in models controlling for other variables (aOR 0.19–0.22, 95% CI 0.13–0.31). The odds of iatrogenic fistula more than doubled in women who reported previous abdominal surgery (aOR 2.48, 95% CI 1.67–3.69), with a more pronounced and statistically significant effect in Tanzania (aOR 2.83; 95% CI 1.80–4.48) than in Malawi (aOR 1.78; 95% CI 0.77–4.08). The odds of iatrogenic fistula increased with increasing parity: aOR 2.36 (95% CI 1.49–3.72) at para 2, aOR 2.58 (95% CI 1.57–4.24) at para 3–5 and aOR 4.20 (95% CI 2.29–7.70) at para 6 or more. Age was not generally associated with increased odds of iatrogenic etiology in this modeling.

A generalized linear model was used to assess the risk difference (0.0467) and 90% confidence interval (− 0.014, 0.107) between the two cadres for iatrogenic fistula following cesarean birth. The 90% confidence interval was entirely contained within the margins of equivalence (− 0.135, 0.135), providing evidence of equivalence between cadres.

## Discussion

Tanzania and Malawi are among the six African countries where associate clinicians perform cesarean sections, particularly in rural areas where the number of doctors is insufficient [16]. This analysis revealed that during the period of 1994–2017, associate clinicians in Tanzania and Malawi were equivalent to physicians in terms of the proportion of iatrogenic fistulas occurring during cesarean birth at an equivalence margin of 13.5%. We are not aware of other evidence on cadre and iatrogenic fistulas following cesarean section from the six countries with cesarean task shifting. Our findings align with reports that use other measures to determine comparability between associate clinicians and physicians attending cesarean births. A review of 1134 complicated births in Tanzanian district hospitals reported no significant differences between assistant medical officers and physicians in terms of outcomes, risk indicators or quality [16]. The results were likewise comparable in Malawi [32], Mozambique [21, 33] and Sierra Leone [17].

Associate clinicians are more likely than physicians to accept and embrace work in rural, remote and underserved areas. In one survey of medical students in six African countries, only 4.8% intended to practice in rural areas, revealing misalignment between medical student intentions and health workforce needs [34]. A study in Mozambique reported that no physicians remained in rural areas 7 years after graduation, in contrast to 88% retention among associate clinicians [35]. The training and deployment of associate clinicians are less expensive than the training and deployment of physicians, so cesarean sections performed by associate clinicians are comparatively less expensive for the health system [36, 37].

Associate clinicians perform the majority of obstetric surgeries in Tanzania and Malawi. According to a survey of 38 district hospitals in Malawi, associate clinicians performed 90% of cesarean sections, 89% of uterine rupture repairs and 60–70% of CS/hysterectomies [32]. According to a survey of 14 hospitals in Kigoma and Mwanza, Tanzania, associate clinicians performed 92% of obstetric surgeries in government hospitals and 73% in mission hospitals [16]. Associate clinicians perform approximately 90% of cesarean sections at district level in Mozambique [21]. In this light, it is unsurprising that associate clinicians performed 86.7% of the cesarean births reported by women who later sought fistula repair. Urogenital fistulas occur most commonly in rural, underserved areas where women do not have sufficient access to emergency obstetric care [38].

Health providers from both cadres attended cesarean births resulting in iatrogenic injury. As previously reported, one-quarter of women with fistulas following cesarean birth had an injury caused by surgery rather than prolonged, obstructed labor [25]. In that analysis, the reported proportions of iatrogenic fistulas after cesarean birth were lower in Tanzania (25.0%) and Malawi (25.9%) than in neighboring countries where associate clinicians do not perform cesarean sections, including Rwanda (26.0%), Kenya (28.8%) and Uganda (30.2%). In contrast to these neighbors, in Tanzania and Malawi the increase in iatrogenic fistulas over time was less than would be expected from the increasing frequency of cesarean section as a mode of birth reported by women who later presented for fistula repair [25].

Although the occurrence of iatrogenic fistulas was lower in cesarean births attended by associate clinicians (24.6%) than those attended by physicians (29.2%), it is not known whether the cadres have fundamentally different cesarean caseloads. One would anticipate that more difficult and complex caseloads would correspond to an increased risk of iatrogenic injury. Associate clinicians are frequently the health professionals available to provide emergency obstetric care in remote, rural locations where physicians are not present.

The proportion of iatrogenic fistulas among women who undergo fistula repair appears to be on the rise in low- and middle-income countries [39]. We previously reported that 26.8% of women with fistulas following cesarean birth had an injury caused by their surgery in a nine-country analysis that included these women in Tanzania and Malawi [25]. Among 229 women with fistulas following cesarean birth in the Democratic Republic of the Congo, 24.0% had iatrogenic injuries [40]. In Ethiopia, a review of 2593 women with fistulas reported that 24.6% had “high bladder fistula (predominantly after surgery.)” [41]. High fistulas similarly accounted for 26.3% (119/452) of vesico-vaginal fistulas in Malawi, “likely due to operative injury rather than obstructed labor.” [42]. Taken together, the evidence supports generalizability of our findings on the proportion of iatrogenic fistulas among women who presented for fistula repair after cesarean birth in Malawi and Tanzania.

Task shifting for emergency obstetric surgeries has long been a subject of investigation [43]. Beyond the comparability of clinical outcomes, compared with physicians, associate clinicians demonstrate superior retention and cost-effectiveness (in training and deployment) [21, 37]. One Mozambican study reported that health workers at all levels expressed satisfaction with the work of associate clinicians, while noting challenges with their professional status and remuneration [44]. Although task shifting to associate clinicians can be an important component in caring for women’s needs for emergency obstetric care, particularly where physicians are scarce, in many countries challenges continue with physicians’ acceptance of associate clinicians providing cesarean sections [22].

Although the adjusted odds ratios for associate clinicians were not statistically significant, it is remarkable that the adjusted odds ratio in Tanzania mirrored the overall (combined) adjusted odds ratio, while the adjusted odds ratio in Malawi differed directionally. This may reflect the smaller sample size in Malawi. It is also possible that physician and associate clinician performance differ between the two countries.

The equivalence margin, δ, is defined as the maximum acceptable difference in iatrogenic fistula occurrence that one is willing to accept in return for the secondary benefits of associate clinicians providing cesarean sections [45]. The equivalence margin in this analysis was set to 13.5%: iatrogenic fistulas following cesarean birth should be no more than 13.5% more frequent in births attended by associate clinicians than in births attended by physicians. At that equivalence margin in this sample of women who presented for fistula repair after cesarean section, associate clinicians were found to be equivalent to physicians in terms of iatrogenic fistula occurrence in cesarean birth.

### Strengths and limitations

Although this retrospective records review included a large number of fistulas following cesarean birth, it is not without limitations. All women in this study developed fistulas and sought treatment, and their experiences may differ from those who died from unattended obstructed labor, women who developed fistulas but did not seek treatment, and women who were spared a fistula through appropriate management of prolonged labor. We did not have records from women who had cesarean births and did not develop fistulas. We do not have a reason to doubt that the included women were representative of all women seeking fistula treatment in the two countries, although selection bias is possible particularly in Malawi, given its smaller sample size. We minimized variability by having a single surgeon confirm the fistula classifications.

We relied on self-reported information from women about their birth experiences, including the condition of their babies. In many cases, years passed between the day of birth and when the woman presented for fistula repair. We acknowledge that women’s recollection of childbirth may differ from how healthcare providers diagnose obstetric problems. To minimize bias, we excluded fistulas that occurred before the start date of our data collection. At the end of our study period, however, it is possible that long-duration fistulas were underrepresented. Given that this review included women who presented for fistula repair after cesarean birth, it may include cesarean sections performed for indications other than prolonged, obstructed labor. As with all retrospective studies, inferences must focus on associations rather than causation.

This review includes more records from Tanzania than Malawi given the differing volumes of fistula repairs conducted by TJIPR, MM, and colleagues. TJIPR’s determination of the cadre attending birth was dependent on his and his colleagues’ knowledge of local facilities and their staffing, which could be subject to recall bias and may be difficult for others to reproduce.

A narrower equivalence margin would provide more definitive evidence of equivalence. Given that only 13% of the included cesarean births resulting in fistulas were attended by physicians, the sample size was insufficiently powered to detect smaller differences between associate clinicians and physicians. Future investigations into the comparability of associate clinicians and physicians may include greater numbers of physician-attended cesarean births, leading to greater statistical power.

Despite these limitations, the equivalence between associate clinicians and physicians attending cesarean births in Tanzania and Malawi provides compelling evidence on the success of cesarean task shifting programs.

## Conclusions

The occurrence of iatrogenic fistulas illustrates the importance of appropriate labor management and cesarean section decision-making, irrespective of the health provider cadre. The high proportion of iatrogenic fistulas among women who present with fistulas after cesarean birth must compel action to improve labor management and surgical safety.

More research is needed to understand associate clinician training, supervision and performance in countries where associate clinicians perform cesarean sections. This can then be compared to medical officer training, supervision and performance in countries where only physicians perform cesarean sections. Such investigations might shape future policy decisions regarding cadre roles. With sufficient evidence, the hallmarks of successful training, supervision and mentorship programs can be applied across cadres and international boundaries.

Given the equivalent performance and the lower cost of training and retaining associate clinicians in rural areas, other countries with insufficient and/or unequally distributed health workforces could consider task-shifting cesarean sections to associate clinicians.

## Data Availability

The data set analyzed during the current study is available from the corresponding author upon reasonable request.
